# Nutrient-stimulated gallbladder emptying is incomplete during critical illness as assessed by 3D ultrasound

**DOI:** 10.1186/2197-425X-3-S1-A285

**Published:** 2015-10-01

**Authors:** MP Plummer, P Kar, CE Cousins, MJ Chapman, T Hausken, KL Jones, M Horowitz, AM Deane

**Affiliations:** University of Adelaide, School of Medicine, Adelaide, Australia; Royal Adelaide Hospital, Intensive Care Unit, Adelaide, Australia; Haukeland University Hospital, Department of Medicine, Bergen, Norway

## Introduction

Gallbladder dysmotility has been implicated as a putative mechanism underlying acute acalculous cholecystitis and lipid malabsorption in the critically ill, despite nutrient-stimulated gallbladder emptying never having been quantified in this population. In health, endogenous cholecystokinin (CCK) stimulates gallbladder emptying.

## Objectives

To determine fasting and nutrient-stimulated gallbladder volumes in critically ill patients.

## Methods

Twenty-four critically ill patients (16M:8F, age 54 ± 16y, BMI 29 ± 6kg/m^2^, APACHE II 17 ± 5, SOFA score 7.5 ± 4, day of admission 5 ± 4) with no history of biliary pathology were compared to 12 healthy volunteers (8M:4F, age 55 ± 19, BMI 24 ± 4). All participants were fasted for 8 hours and had a feeding catheter inserted into the duodenum. At T = -30min 3D images of the gallbladder were acquired and wall thickness and presence of sludge recorded. Between T = 0-120min 20% intralipid was infused into the duodenum at 2kcal/min. Ultrasound measurements of the gallbladder were obtained at 30min intervals until T = 180min. Ejection fraction (%) and the change in gallbladder volume (mls) were calculated from T = 0-120min. Blood samples were taken at 30 minute intervals for the measurement of plasma CCK. Differences between groups were analysed using Student's t-test or Mann-Whitney test as appropriate and data are presented as mean (SD) or median [IQR]. Unadjusted correlation analyses were performed for demographic, ultrasound and CCK data.

## Results

The cohorts were well matched for age, but BMI was greater (P < 0.01) in the critically ill. In the critically ill, fasting gallbladder volumes were greater (by about three fold) [ICU 60[38-90] vs health 22[15-24]ml; P < 0.001], as was wall thickness [0.44(0.14) vs 0.26(0.08)mm; P < 0.001]. Sludge was evident in the majority (71%) of patients but in none of the healthy participants. The ejection fraction was less in the critically ill [50[10-82] vs 77[72-82]%; P = 0.01], but there was no difference in the change in gallbladder volume [22[11-32] vs 16[12-20]ml; P = 0.18]. Gallbladder volumes post lipid infusion (T = 120min) were much greater in the critically ill [22[9-66] vs 4[3-6]ml; P < 0.01]. There was no difference in fasting CCK concentration [ICU 5.3(2.5) vs health 4.7(1.2)pmol/L; P = 0.5] or the incremental increase in plasma CCK T0-120 min [5.8(3.8) vs 4.6(3.7)pmol/L; P = 0.37]. There were no associations between fasting CCK concentration and fasting gallbladder volume, and CCK increment and gallbladder ejection fraction in the critically ill. There was an association between severity of illness score and gallbladder ejection fraction [r = -0.5; P < 0.05].

## Conclusions

When fasted, critically ill patients appear to have larger and thicker walled gallbladder than healthy subjects. In response to nutrient stimulation the change in gallbladder volume is unaffected, but the ejection fraction is less leading to incomplete emptying.

## Grant Acknowledgment

Study supported by a NHMRC Postgraduate Scholarship.

